# Differentiating Pemphigus Foliaceus From Pemphigus Vulgaris in Clinical Practice

**DOI:** 10.7759/cureus.17889

**Published:** 2021-09-11

**Authors:** Natalie Stumpf, Simo Huang, Lawrence D Hall, Sylvia Hsu

**Affiliations:** 1 Department of Dermatology, Temple University Hospital, Philadelphia, USA; 2 Department of Dermatopathology, Institute for Dermatopathology, Dermpath Diagnostics, Newtown Square, USA

**Keywords:** autoimmune bullous dermatoses, enzyme-linked immunosorbent assay (elisa), direct immunofluorescence, pemphigus vulgaris, pemphigus foliaceus

## Abstract

Pemphigus is a skin condition that causes intraepidermal separation of keratinocytes. Multiple types of pemphigus exist, including pemphigus vulgaris and pemphigus foliaceus. These can be differentiated by histopathology, clinical presentation, appearance of lesions, and antibodies, among other factors. It is important to distinguish between the two because of differences in management and prognosis. Here we present a case of pemphigus foliaceus, as well as a discussion of the key differences between pemphigus foliaceus and vulgaris.

## Introduction

Pemphigus describes a family of dermatoses that causes intraepidermal acantholysis, or separation of keratinocytes [1,2]. Both pemphigus vulgaris (first described in 1777) and pemphigus foliaceus (first described in 1844) are autoimmune conditions involving antibodies to desmogleins, which are transmembrane glycoproteins that form part of the desmosome [1,2]. Pemphigus vulgaris is most commonly seen in individuals around age 40 to 60 years. In contrast, pemphigus foliaceus affects a broader age range [3].

## Case presentation

**Figure 1:**
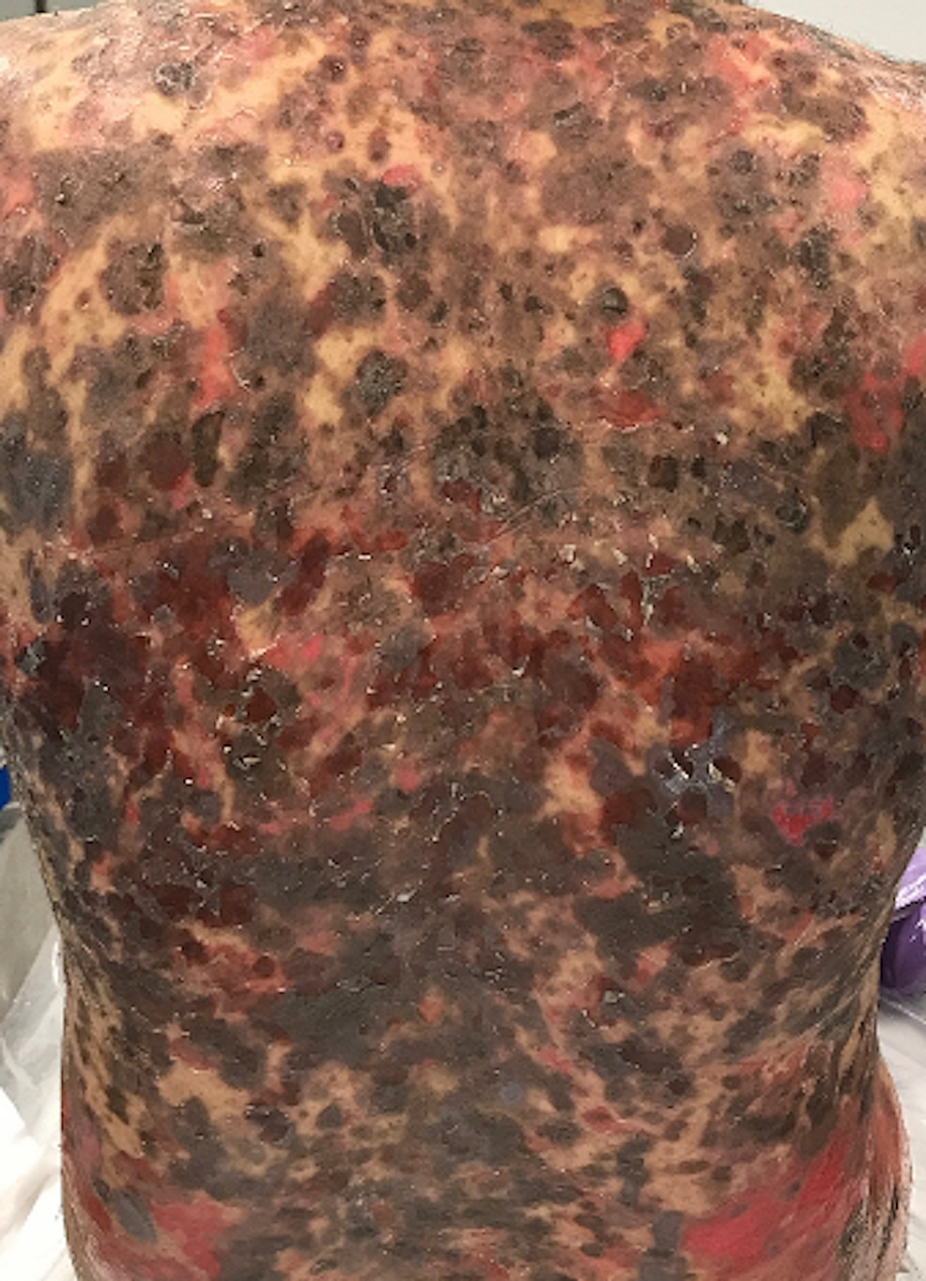
Clinical image on presentation.

**Figure 2:**
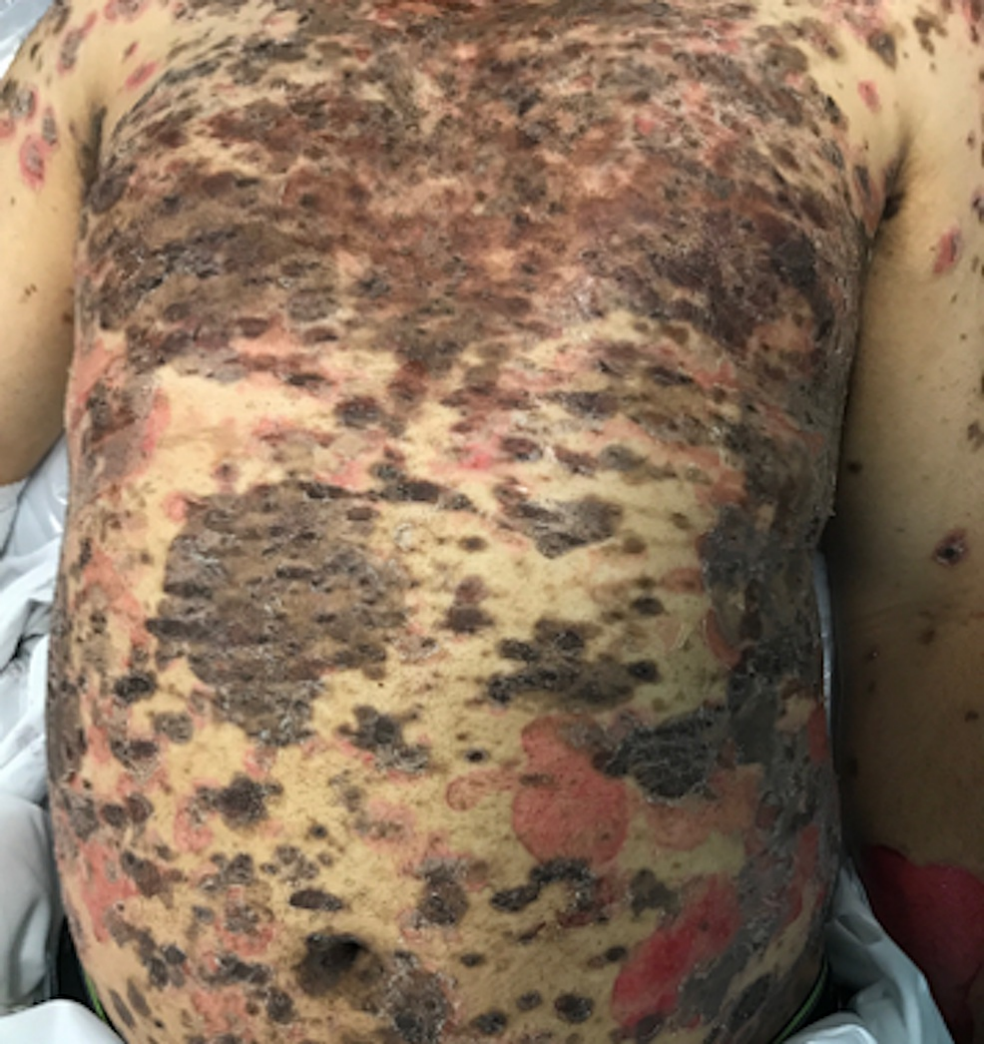
Clinical image on presentation.

**Figure 3:**
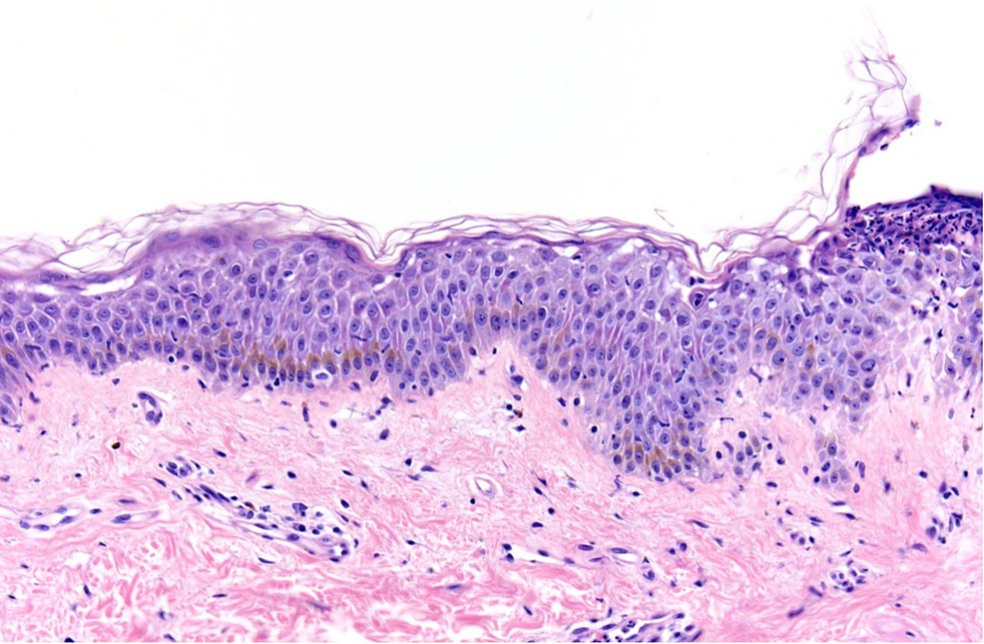
Subcorneal acantholysis with subcorneal neutrophilic infiltrate and sparse superficial perivascular lymphocytic inflammation (hematoxylin and eosin, 20x).

A 39-year-old male presented to the clinic with an eight-month history of a painful rash on his face and upper body, covering a total body surface area (BSA) of 40%. On examination, the patient had crusted plaques with extensive erosions on his scalp, face, trunk, arms, and thighs (Figures 1, 2). There was no involvement of the mucosal membranes. A skin biopsy with hematoxylin and eosin (H&E) staining showed subcorneal acantholysis with neutrophils in the upper epidermis and rare necrotic keratinocytes (Figure 3). Direct immunofluorescence (DIF) showed intercellular immunoglobulin deposition consistent with pemphigus foliaceus. Enzyme-linked immunosorbent assay (ELISA) was performed for desmoglein 1 (Dsg1) and desmoglein 3 (Dsg3). His Dsg1 titer was positive at a level of 183.7, and Dsg3 was negative.

Prednisone was started for suspicion of pemphigus foliaceus. The results of the H&E biopsy, DIF, and ELISA were confirmatory. A thorough history did not reveal any incriminating medications. On a two-week follow-up, an exam of the face showed diffuse peeling with more hyperpigmented patches and fewer active lesions. Exam of the arms, back, and chest showed re-epithelialized erosions. Prednisone was continued at a tapering dose.

## Discussion

Our patient initially had an outside skin biopsy that was read as pemphigus vulgaris with focal features of pemphigus foliaceus. Careful analysis of the clinical presentation, as well as further pathologic and serologic studies, pointed instead to pemphigus foliaceus, as summarized in Table 1.

**Table 1:** Diagnostic features of pemphigus vulgaris and pemphigus foliaceus. Dsg, desmoglein; IgG, immunoglobulin G.

	Pemphigus vulgaris	Pemphigus foliaceus
Antibodies	Dsg3, +/−Dsg1	Dsg1 only
Histopathology	Suprabasilar acantholysis	Subcorneal acantholysis
Direct immunofluorescence	Intercellular IgG	Intercellular IgG
Indirect immunofluorescence	Monkey esophagus	Guinea pig esophagus
Area of involvement and distribution	Mucosal if positive for Dsg3 only; mucocutaneous if positive for both Dsg3 and Dsg1	Skin; does not involve mucosal membranes; starts with seborrheic distribution that may become generalized
Appearance of lesions	Erosions on mucosa, flaccid blisters, and erosions on skin	Crusted erosions without intact blisters

It is important to distinguish pemphigus foliaceus from pemphigus vulgaris due to the difference in management and prognosis. Treatment for both pemphigus vulgaris and pemphigus foliaceus involves oral corticosteroids with the addition of steroid-sparing agents; however, pemphigus foliaceus tends to respond better and requires a shorter duration of therapy [4]. Prednisone or prednisolone is often used. Steroid-sparing agents include azathioprine, mycophenolate mofetil, rituximab, or cyclophosphamide. Intravenous immunoglobulin (IVIg) may also be used. Nonpharmacologic treatment for the refractory disease may involve immunoadsorption or plasmapheresis [4]. Superpotent topical corticosteroids may be considered for pemphigus foliaceus that remains localized [5].

Pemphigus vulgaris is usually positive for antibodies to Dsg3, resulting in suprabasal acantholysis of mucous membranes [6]. Mucocutaneous pemphigus vulgaris shows antibody positivity to both Dsg3 and Dsg1 [7]. In contrast, pemphigus foliaceus is positive for Dsg1 and negative for Dsg3. Dsg1 antibodies are associated with superficial acantholysis and a subcorneal split [8]. Drug-induced pemphigus, which has been reported with penicillamine and captopril, is more likely to be pemphigus foliaceus. In a review of cases of drug-induced pemphigus, ELISA titers were positive for Dsg1 in roughly 70% of cases compared to Dsg3 in roughly 35% [9].

On clinical presentation, pemphigus vulgaris usually starts with oral erosions. Patients may go on to develop flaccid bullae anywhere on mucocutaneous sites. As bullae rupture, crusted lesions form that heal with hyperpigmentation [5]. In contrast, pemphigus foliaceus has a seborrheic distribution at onset, favoring the face, scalp, and upper trunk. It usually spares the oral mucosa [4]. Superficial bullae rapidly form crusted erosions, which are the predominant lesion.

The difference in morphology and distribution between pemphigus vulgaris and pemphigus foliaceus is explained by the compensation theory. Dsg1 is expressed throughout the epidermis, but at a higher concentration in the superficial layers, whereas Dsg3 expression favors the basilar epidermis. Dsg3 is the predominant desmoglein expressed in mucous membrane epithelium. Dsg1 autoantibodies induce superficial bullae due to Dsg3 compensation in the deeper epidermis. Conversely, Dsg3 autoantibodies result in mucosal erosions because Dsg1 compensates at cutaneous sites. Mucocutaneous eruptions occur only when both autoantibodies are present [5].

On histopathology, pemphigus vulgaris demonstrates suprabasilar intraepidermal acantholysis with dermal eosinophils. Keratinocytes form a “tombstoning” pattern due to their adherence to the hemidesmosome. Pemphigus foliaceus shows subcorneal acantholysis with the split occurring within the granular layer [5]. Acantholysis can involve the hair follicles in both disorders. Direct immunofluorescence is the most sensitive test for pemphigus. DIF patterns are similar in both vulgaris and foliaceus and demonstrate intercellular immunoglobulin G (IgG) deposition in a chicken wire/honeycomb pattern; for vulgaris, the sensitivity is 100% [4]. Indirect immunofluorescence using the substrates listed in Table 1 shows intercellular staining [1]. The diagnosis of vulgaris vs. foliaceus can be further determined by using ELISA to detect the target antigen (Dsg3 vs. Dsg1). ELISA is both highly sensitive and specific, with sensitivity up to 100% for untreated pemphigus and specificity up to 98% reported in some studies [10]. See Table 1 for a summary.

## Conclusions

Pemphigus foliaceus and pemphigus vulgaris both belong to the pemphigus family but have different clinical presentations including the appearance of lesions and the areas of involvement. ELISA testing for the target antigen is highly specific and sensitive; testing will reveal Dsg3 in pemphigus vulgaris (+/−Dsg1), whereas only Dsg1 will be positive in pemphigus foliaceus. These nuances are important to elucidate because of their significance in terms of treatment and prognosis, as pemphigus foliaceus can require a shorter duration of therapy.

## References

[1] Schmidt E, Kasperkiewicz M, Joly P (2019). Pemphigus. Lancet.

[2] Kayani M, Aslam AM (2017). Bullous pemphigoid and pemphigus vulgaris. BMJ.

[3] Habif TP, Dinulos J, Chapman MS, Zug K (2017). Skin Disease Diagnosis and Treatment. https://www.elsevier.com/books/skin-disease/habif/978-0-323-44222-0.

[4] Melchionda V, Harman KE (2019). Pemphigus vulgaris and pemphigus foliaceus: an overview of the clinical presentation, investigations and management. Clin Exp Dermatol.

[5] Bolognia J, Schaffer J, Cerroni L (2017). Pemphigus. Dermatology.

[6] Ruocco V, Ruocco E, Lo Schiavo A, Brunetti G, Guerrera LP, Wolf R (2013). Pemphigus: etiology, pathogenesis, and inducing or triggering factors: facts and controversies. Clin Dermatol.

[7] Bystryn JC, Rudolph JL (2005). Pemphigus. Lancet.

[8] Hans-Filho G, Aoki V, Bittner NR, Bittner GC (2018). Fogo selvagem: endemic pemphigus foliaceus. An Bras Dermatol.

[9] Ghaedi F, Etesami I, Aryanian Z (2021). Drug-induced pemphigus: a systematic review of 170 patients. Int Immunopharmacol.

[10] Harman KE, Gratian MJ, Seed PT, Bhogal BS, Challacombe SJ, Black MM (2000). Diagnosis of pemphigus by ELISA: a critical evaluation of two ELISAs for the detection of antibodies to the major pemphigus antigens, desmoglein 1 and 3. Clin Exp Dermatol.

